# Understanding Color Associations and Their Effects on Expectations of Drugs’ Efficacies

**DOI:** 10.3390/pharmacy10040082

**Published:** 2022-07-13

**Authors:** Rema M. Amawi, Michael J. Murdoch

**Affiliations:** 1 Sciences and Liberal Arts Department, Rochester Institute of Technology, Dubai 345019, United Arab Emirates; 2 Munsell Color Science Laboratory, Rochester Institute of Technology, Rochester, NY 14623, USA; mmpocs@rit.edu

**Keywords:** pill color, color association, drug efficacy, color perception, cultural differences

## Abstract

Colors influence our daily perceptions and expectations that manifest in a variety of ways. This research has three main objectives: to demonstrate the relationship between the colors of pills and their expected efficacies, to test this effect on a wide variety of demographics, thereby demonstrating their influence on choices made by participants. Finally, to understand the reasoning behind the choices made by participants, and the color associations exhibited. The results of a series of surveys showed clear similarities and differences across various demographics. The strongest and most consistent color associations were those of white with pain relief and red with stimulant efficacies. The color associations found were red with aggression and power, blue with calmness and serenity, white with calm and purity, yellow with energy, and green with environment and health. The findings of this study can help pharmaceutical companies, and medical practitioners, to better make, market, and prescribe pills, depending on the geographical location, ethnicity, and age group of the patient. This may also strengthen the perceived effects of the pills on patients overall by increasing their compliance rates.

## 1. Introduction

Color is an integral part of our daily lives, and affects the decisions we make throughout the day: from the clothes we choose to wear, the places we go to, the things we buy, and on top of all, what we do based on how we feel. Research has shown that color has different meanings for a variety of demographics such as geographical locations, age groups, and gender [1,2]. These meanings are based on associations that can be formed and/or learnt in various stages of one’s life, and may change from one stage to the other [3]. Color, referred to as the emotion messenger by Ou in 2012, has different effects on people’s perception and expectation. Perception can be defined as the initial or intuitive interpretation of a stimuli based on the gathered sensory information, while expectation is an opinion formed about something that may materialize based on perception. Interest in this area has grown over the years and researchers have referred to it in a variety of ways such as color meaning, color image, color emotion, and expectations [4]. Prior research done by Jonauskaite et al. focused on differentiating between the feelings associated with color, and the feelings caused by color [5].

Colors are sometimes categorized as warm or cool. A study done by Torres et al. [6] on the effect of warm colors versus cold colors relied on valence and arousal effects, where their findings demonstrated that the effects of warm colors in room interiors promoted higher arousal levels, versus the effects of cold colors in room interiors which promoted lower arousal levels in relation to both genders.

Color is also a key characteristic in identifying medications [7]. Leslie, as early as 1954, related the effect of color on placebo drugs [8]. Research continued and included Lasagna et al. in 1958 who tested the effect of yellow color of placebo pills on patients and their association with increasing energy effects [9]. Patients reported an increase in energy levels. In 1970, Schapira et al. found that green-colored tablets were best suited for improving anxiety symptoms, and yellow-colored tablets were best suited for depressive symptoms [10]. Blackwell et al. in 1972 tested blue and pink placebo drugs, and found that blue tablets had a drowsier effect than pink ones [11]. In the 80’s, Buckalew and Coffield, as cited by Bhugra, tested the same effects across several ethnicities, and stated that Caucasians perceived white-colored pills as analgesics, and African-Americans perceived them as stimulants. Furthermore, the expectations were reversed for black-colored pills [12]. In 2015, Wan et al., reported that red-colored pills have the highest alerting effect, while white-colored pills to be best suited for combating headaches, based on participants’ choices. Tao et al. followed in 2017, and did a comparison across cultures. Their findings were that red was being perceived as a stimulant across cultures, and blue as a depressant. Tao et al. did another study in 2018 and reported black and yellow-colored pills being perceived as hallucinogens, white, blue, and green-colored pills being perceived as depressants, while red-colored pills as having stimulant effects. In both studies by Tao et al., it was evident that gender had an effect on certain colors such as blue and black. Furthermore, some perceived expectations can change over time for some colors, and that is why it is important to consider age as an influential factor as well. For example, blue-colored pills used to be considered as a sedative, and after the introduction of the Viagra pill in 1998, the perception of the blue-colored pill changed to having a stimulating effect instead [13]. Additionally, white-colored pills were perceived as the least effective, but more recently, they are perceived as highly effective in treating headaches due to their association with Aspirin [14].

The efficacies and colors examined were chosen based on common ones previously studied by researchers such as Craen et al. in 1996, and Tao et al. in 2017 and 2018. While research in the field of pill colors continues to provide evidence of the color association with human perception and emotions, along with the effects on the expected efficacy of pills, more research is needed at a more diverse level to better understand the basis behind the emerging patterns, and their relation to people’s demographics.

The objective of this research was to study the following: (1) pill colors affect peoples’ perception and expectation of the efficacy of drugs, (2) whether and how the color association of the pill can be influenced based on participants’ demographics, and (3) understand the reasoning behind the color associations. Two separate experiments were conducted at Rochester Institute of Technology (RIT)’s global locations, from which conclusions were drawn from the aggregated results.

## 2. Experiment 1

### 2.1. Method

The first experiment was launched at RIT’s global campuses in the USA, UAE, Croatia, and Kosovo. Participants were asked to complete an online interactive survey, and were requested to agree to the terms and conditions on a consent form, register using their RIT credentials, and to provide their demographics information (see Table 1). It is worth noting that this study builds on our previous work [15], and the results from both studies were aggregated for the analysis.

**Figure 1:**
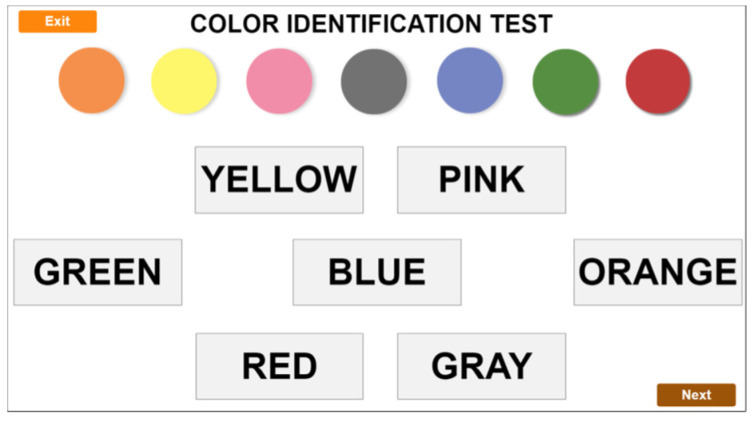
Color Identification Test taken by participants.

**Figure 2:**
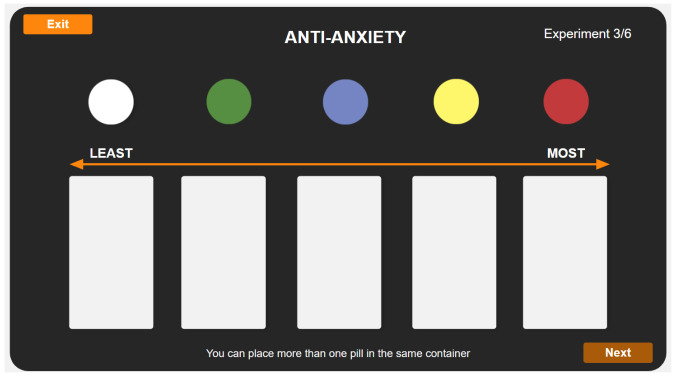
Experiment screen where participants rated colored-pills.

**Figure 3:**
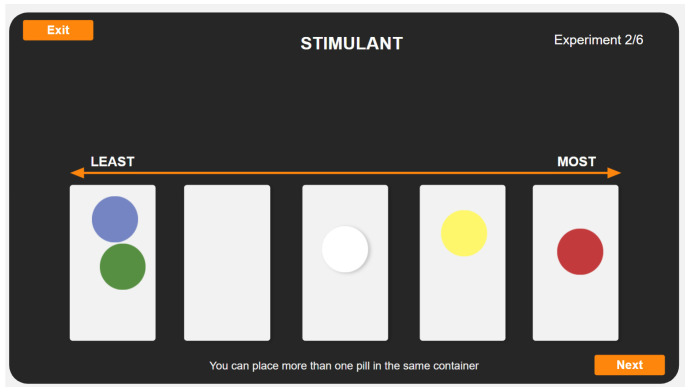
Participants can place more than one pill at each location.

Participants performed a color identification test, and then performed six experimental tasks: one for each perceived efficacy: sedative, stimulant, anti-anxiety, pain relief, antacid or hallucinogenic. In each efficacy-specific task, participants arranged five pill-shaped colors (blue, green, red, white, and yellow) on a scale of LEAST (Score = 1) to MOST (Score = 5) effective, using a drag-and-drop interface. Multiple pills could be placed at any location and would score the same, as it was a categorization task, not a rank-order task (See Figure 1, Figure 2 and Figure 3).

#### Color Stimuli

**Figure 4:**
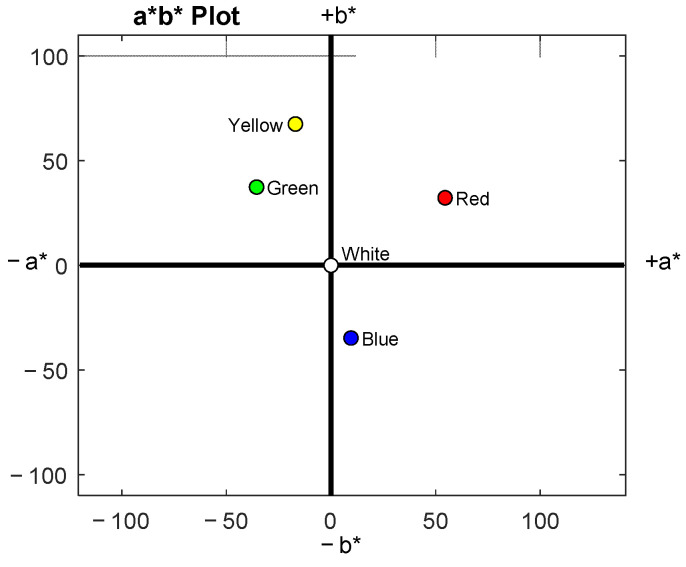
Pill colors on CIELAB a* b* Plot.

The sRGB values and CIELAB coordinates for the chosen pill colors are listed in Table 2, and plotted in Figure 4.

### 2.2. Results

Out of 648 participants, only 615 records were retained due to incomplete data, or the exclusion of results by participants who failed the color identification test. Given the similarity between this experiment and our previous research [15], the data for UAE and USA were combined prior to conducting the analysis. The responses breakdown by demographics can be found in Table 3. The data collected was ordinal in nature, with a non-normal distribution; therefore, a non-parametric analysis was performed. Kruskal-Wallis (KW) statistical tests were initially performed in order to test whether color had a significant effect on the expectation of efficacy for each of the pill categories. Following significant KW results, Mann–Whitney, Wilcoxon rank-sum tests were used to check pairwise comparisons of all of the pill colors. Holms-Bonferroni corrections were used, and Cohen’s d effect sizes were computed and then interpreted using Sawilowsky’s categorization [16]. Next, the data were split by the demographics ethnicity, age, and location for every separate efficacy, and the same analysis used. These three demographics were given priority because they may be more directly addressed through development and marketing strategies. Mean rank values computed with KW were divided by sample size and plotted in the following figures. Higher rank values correspond to higher ratings of perceived expected efficacy.

#### 2.2.1. Overall Effect of Color by Efficacy

The Kruskal-Wallis tests revealed that pill colors had a significant effect on perceived efficacy for all efficacy categories, with Chi-square and *p*-values as follows: sedative, X^2^(4) = 234.15, *p* < 0.001; stimulant, X^2^(4) = 438.55, *p* < 0.001; anti-anxiety, X^2^(4) = 635.5, *p* < 0.001; pain relief, X^2^(4) = 294.85, *p* < 0.001; antacid, X^2^(4) = 201.51, *p* < 0.001; and hallucinogenic, X^2^(4) = 185.93, *p* < 0.001. Mann–Whitney, Wilcoxon rank-sum post hoc test results are shown in Table 4.

**Figure 5:**
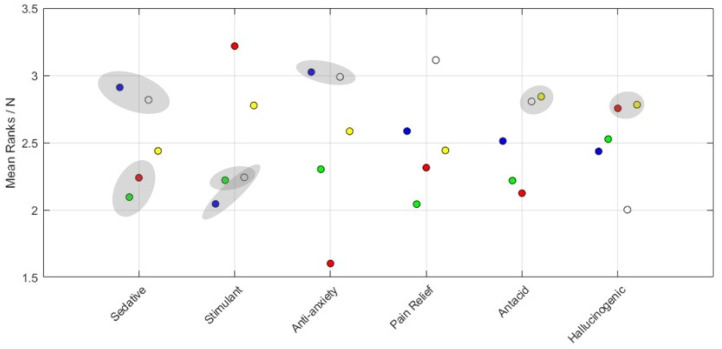
Ranking values for expected efficacy. Gray regions contain colors which are not significantly different from one another.

Figure 5 illustrates the resulting perceived efficacy, using shaded ellipses to connect colors that were found not significantly different from one another. For sedatives, blue and white were ranked significantly higher than the remaining colors, but not different from one another. Yellow ranked higher than the group red and green. In the stimulant category, red ranked significantly highest, followed by yellow, which ranked significantly higher than the rest. For the anti-anxiety category, blue and white were found significantly highest, followed by yellow, green, and red, respectively. For pain relief, all colors were significantly different, highest to lowest: white, blue, yellow, red, then green. Yellow and white ranked significantly highest for the antacid efficacy. Blue was ranked significantly higher than green, which was followed by red. Lastly, yellow and red together ranked highest for the hallucinogenic category, followed by green, blue, then white.

#### 2.2.2. Effect of Color and Ethnicity

For every ethnicity, KW tests were conducted to assess the expected effect of pill colors, as listed in Table 5. Results revealed that color has a significant effect on the expected sedative efficacy for Asians, Europeans, and North Americans. In the stimulant category, except for South Americans, color was significant for all ethnicities, while all but Africans found color significant for anti-anxiety. As for pain relief, the results of Asians, Europeans, Middle-Easterners, and North Americans revealed color had a significant effect. The results of all ethnicities showed a significant effect for antacid, and all groups with the exception of South Americans revealed that color had a significant effect for the hallucinogenic category.

**Figure 6:**
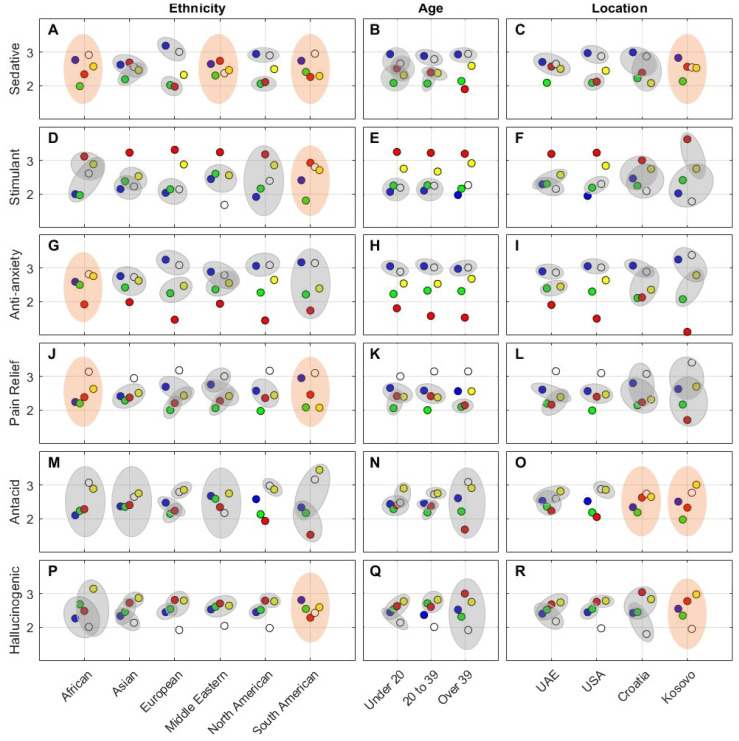
Each subplot (**A**–**R**) shows the rank order values for each pill color, broken down by ethnicity, age, location, and by efficacy. Orange-shaded areas denote non-significant Kruskal-Wallis results. Gray-shaded areas contain colors which have no significant differences between them.

Post-hoc tests followed each significant KW test result, and the resulting values are listed in Table A1 in the appendix. Plots in the left column of Figure 6 visualize the relationships between the colors by ethnicity for every efficacy. As in the previous figure gray-shaded regions indicate non-significant pairwise differences; additionally, non-significant KW results are indicated using orange ellipses. The non-significant KW results are primarily seen with the smallest demographic groups: Africans (N = 32) and South Americans (N = 18). The results in Figure 6 for ethnicity mostly follow the overall results in Figure 5, with some minor re-ordering in some cases.

#### 2.2.3. Effect of Color and Age

As with ethnicity, KW tests were used to assess each age bracket by efficacy, and the results are listed in Table 6. Post-hoc test results are provided in Table A2 in the Appendix A. Color had a significant effect on the perceived efficacy for all efficacies from all age groups, and the data are visualized in the center column of Figure 6. Again, the overall patterns seen in Figure 5 are consistently found in all age groups, though it may be observed that the dominance of white for pain relief (see plot K) gets even stronger from younger to older groups. Similarly, the weakness of red for anti-anxiety (see plot H) seems to get more pronounced from younger to older.

#### 2.2.4. Effect of Color and Location

Location was also tested using KW tests for each location and efficacy, and results are presented in Table 7. The data revealed pill color has a significant effect on all efficacies for UAE and USA participants. However, non-significant results were found for antacid for participants from Croatia, and sedative, antacid, and hallucinogenic categories for participants from Kosovo. The right column of plots in Figure 6 visualize these results, and post-hoc test results are given in Table A3 in the Appendix A. The main difference from each of these plots and the overall results in Figure 5 appears to be the relative strength of the rank ordering.

**Table 7:** KW statistical test results for each campus location and efficacy category.

	Sedative		Stimulant		Anti-Anxiety		Pain Relief		Antacid		Hallucinogenic	
Location	X^2^	*p*	X^2^	*p*	X^2^	*p*	X^2^	*p*	X^2^	*p*	X^2^	*p*
UAE	23.2	**0.0001**	67.67	**<0.001**	64.92	**<0.001**	64.96	**<0.001**	19.48	**0.0006**	19.96	**0.0005**
USA	222.82	**<0.001**	358.77	**<0.001**	540.66	**<0.001**	209.82	**<0.001**	189.46	**<0.001**	143.64	**0.0000**
Croatia	16.19	**0.0028**	13.76	**0.0081**	19.76	**<0.001**	16.47	**0.0024**	5.53	0.2372	22.65	**0.0001**
Kosovo	3.21	0.523	27.05	**<0.001**	46.24	**<0.001**	21.13	**0.0003**	8.24	0.0831	8.15	0.0862

Red text indicates significant effects (alpha ≤ 0.05).

### 2.3. Discussion

Color, and its relations with demographics, did have a significant effect as evident in the results of this experiment (See Figure 5) as the expected efficacy of different colored pills are very different. One caveat from the study is that the efficacy categories were always presented to participants in the same order, which means any order effect that might have been present was not controlled during the experiments. The following is a summary by color: red resulted in some of the strongest differences, ranking highest overall for the stimulant efficacy category and the lowest overall for the anti-anxiety efficacy category (See Figure 5). These findings match the results from all prioritized demographics (See Figure 6). This is also aligned with our findings from our first study [15], and previous literature, where red had been repeatedly associated with activity and stimulation, and increased levels of anxiety and excitement [17,18,19,20,21,22,23,24].

Blue and white colors, often together, ranked the highest for sedative and anti-anxiety efficacy categories, following expectations because blue was associated with sedatives [13,22], calmness, quiet, water, and sky [5,19,20,25,26,27,28], while white was associated with calm, peace and gentle [27,29]. This is also aligned with the results of most ethnicities, all age groups and geographical locations.

White-colored pills are most associated with the pain relief efficacy, as previously discussed [15], and its associations with commonly known medications [12,14,23,24] as well as the color itself being associated with relief as reported by Jonauskaite in 2020 [5]. Similarly, there was a consensus among all prioritized demographics on ranking white as a highly effective color for the pain relief efficacy.

Yellow and white-colored pills were found highest ranked for the antacid efficacy category, and that can also be associated with commonly known medications in the market being white in color, such as Rennie, Gaviscon, and Alka-Seltzer.

Yellow and red-colored pills were found highest ranked for the hallucinogenic efficacy but not significantly different from each other. The associations of the yellow-colored pills are supported by our previous study [15] and prior work done by Tao et al. in 2017 and 2008, as well as others. Hallucinations are mainly perceptions created by the mind. However, the color yellow is associated with activity, stimulation, joy and amusement [5,20].

It is worth noting that green did not have any strong associations to any of the efficacies, except for being ranked least effective compared to all other colors for pain relief. Green is commonly associated with health, nature, fresh, and grass [5,27,28].

## 3. Experiment 2

### 3.1. Method

**Figure 7:**
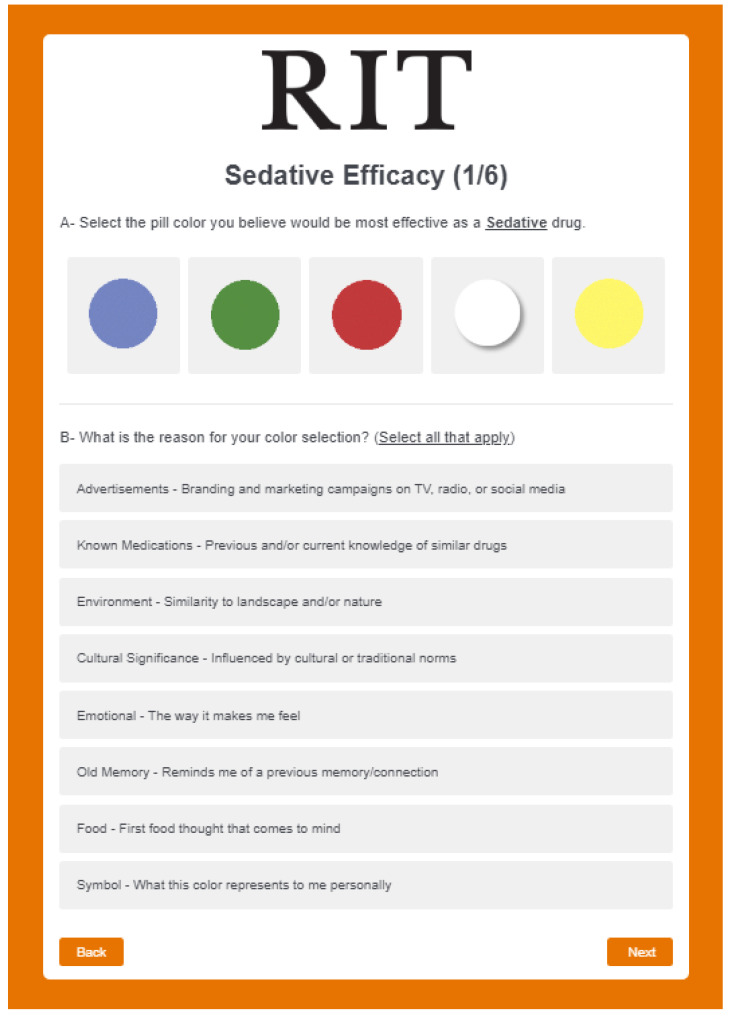
Experiment 2—Part 2A: color selection and reasoning.

The second experiment was launched at RIT’s global campuses in the USA, UAE, Croatia, Kosovo and China. Participants were asked to complete an online interactive survey in a similar fashion to the first experiment with the same color stimuli and efficacies. In this experiment, participants started by performing a color identification test, and if successful, were able to proceed to Part 2A: Reasoning. This part was divided into six tasks: for each efficacy category, participants selected a single color they perceived to be most effective for that particular efficacy, and then selected one or more reason(s) behind their choice from a pre-defined list (see Table 8). This task was repeated for each of the six efficacies (See Figure 7).

**Figure 8:**
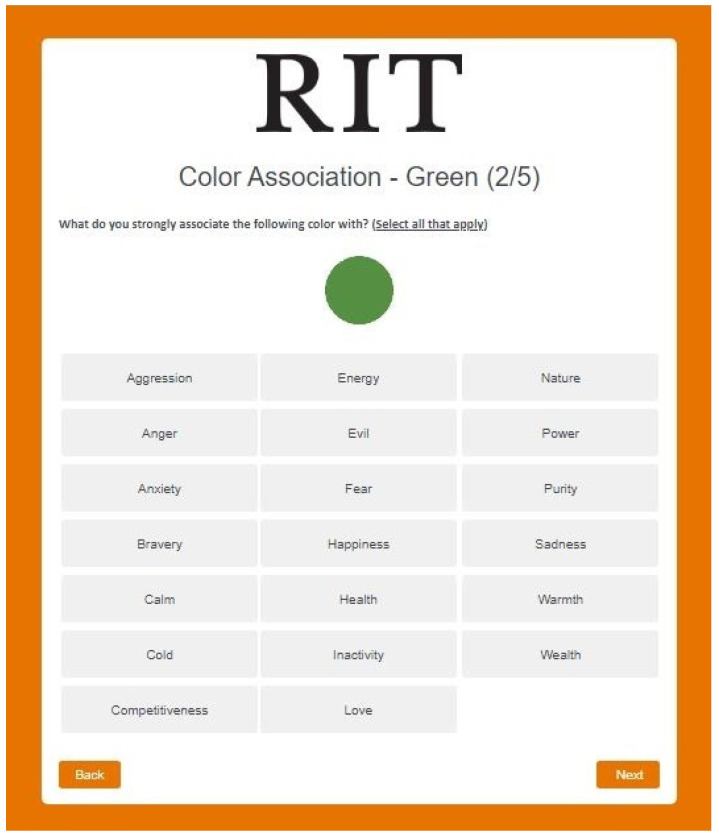
Experiment 2—Part 2B: color association.

After completing Part 2A, participants proceeded to Part 2B: Color Association (Figure 8), where they were presented with one color at a time, and asked what do they strongly associate the color with from a pre-defined list.

### 3.2. Results

410 responses were analyzed out of the original set received from 560 participants who took the survey, excluding incomplete results and responses from participants who failed the color identification test. Response rates in percentages were used to rate each color. The breakdown of by demographics is in Table 9.

**Figure 9:**
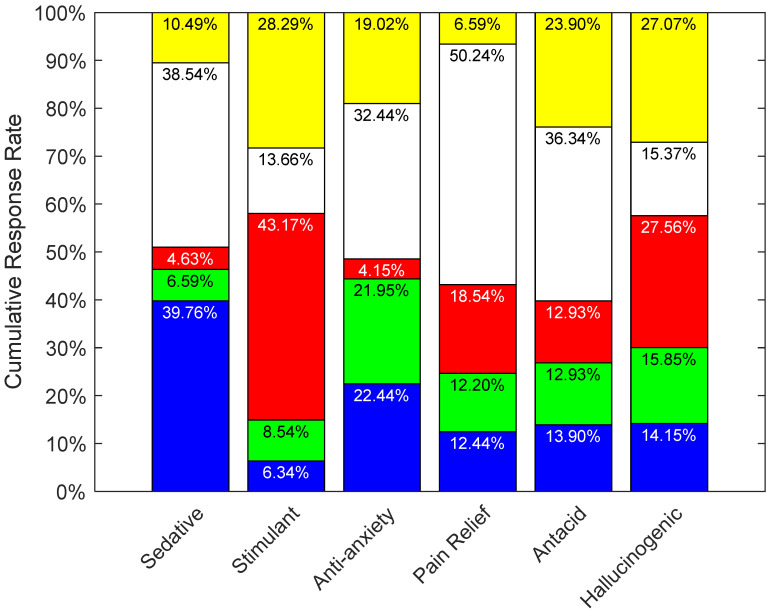
Participants’ response rates for colors most strongly associated with each efficacy. Chi-square goodness of fit tests found all of these frequency distributions are significantly different from random chance.

Figure 9 shows the observed frequency distributions. Each was tested with a chi-square goodness of fit test against a random, uniform distribution, and each was found to be significantly different (*p* < 0.001). For the sedative efficacy (See Figure 9), 39.76% of participants selected blue, followed closely by 38.54% who chose white. Red was the lowest at 4.63% response rate. For the stimulant efficacy, red strongly scored the highest with a 43.17% response rate, followed by yellow at 28.29%. On the other hand, blue scored the lowest at 6.34%, which is understandable, given that it is expected to have the opposite effect of a sedative. As for anti-anxiety, white was the top choice of participants with a 32.44% response rate, while red scored the lowest with a 4.15%. Participants chose white as their top choice for both the pain relief and antacid efficacies with a 50.24% and 36.34% response rates respectively. While yellow scored the lowest response rate of 6.59% for pain relief, it scored the second highest for antacid with 23.90%. Both green and red scored the lowest for antacid at 12.93% equally. As for the hallucinogenic efficacy, red scored the highest at 27.56% and yellow was closely behind at a 27.07% response rate. Notably, these results correlate to those obtained in experiment 1 (See Figure 5).

#### 3.2.1. Part 2A: Reasoning

**Figure 10:**
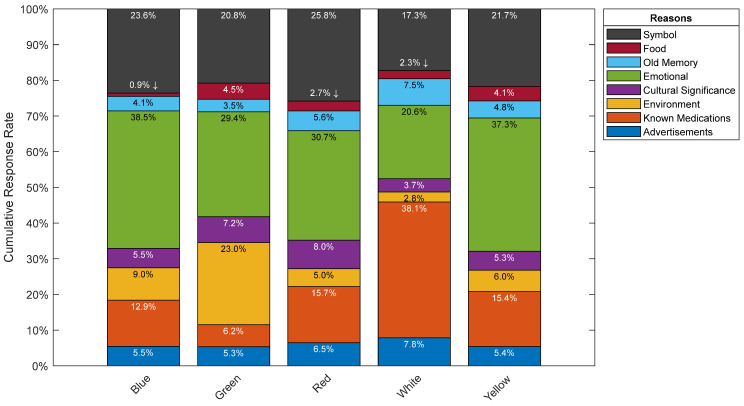
Participants’ response rates showing overall reasoning by color.

**Figure 11:**
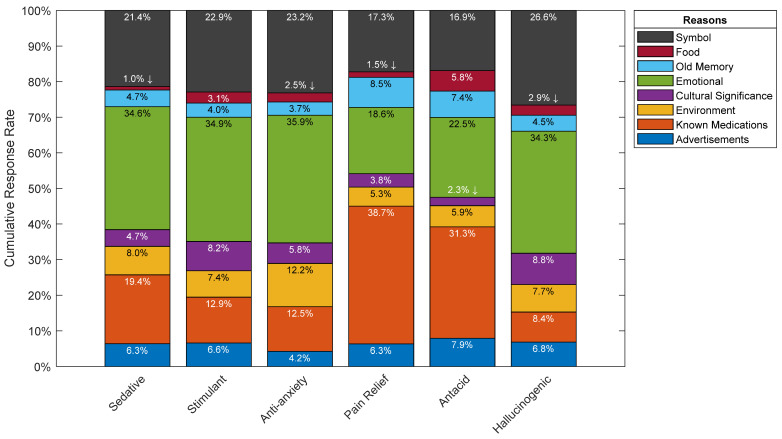
Participants’ response rates showing overall reasoning by Efficacy.

**Figure 12:**
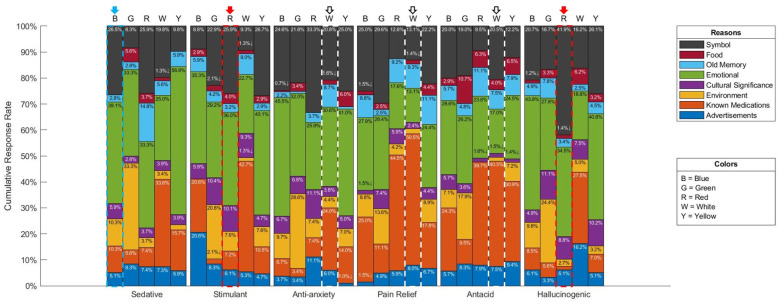
Participants’ response rates showing overall reasoning by Efficacy-Color combination. Arrows indicate the top color choice for each efficacy.

Overall, participants’ justifications for their color choices across all efficacies irrespective of the color can be attributed to either an emotional reaction, a symbolic reason, or known medications where these reasons scored the highest by far (See Figure 10 and Figure 11). It is interesting to note that advertising had no meaningful impact on participants’ color choices, where it scored among the lowest between the various reasons provided by participants. Results by efficacy-color combination are shown in Figure 12, where 33.3% of participants attributed their choice of green for the sedative efficacy to environmental reasons. This could be due to the association of green to herbal remedies such anise and chamomile, which do have sedative effects. Furthermore, advertisements scored 20.6% for blue as a stimulant, which may be attributed to the popularity of Viagra, the blue pill, and its abundant media coverage. Another interesting choice of color per efficacy is the choice of green, and relating it to the environment for the anti-anxiety efficacy where it scored 28.6%, and this could be due to the calming effect of the natural environment. Finally, for the hallucinogenic efficacy, the symbolic reasoning scored the highest for red at a 41.9% response rate, and this could be attributed to the most commonly known hallucinogenic: the poisonous mushroom, *Amanita phalloides*, which happens to be mainly red in color. It could also be attributed to the red pill example from the Matrix movie, associated with learning deeper truths in “Wonderland”. It is worth nothing that such inferences rely on cultural references and have been primarily derived from western cultures.

In Figure 12, and with reference to the sedative efficacy, blue and white were identified as the top choices for participants. The majority of those who chose blue for the sedative efficacy, made that choice because of an emotional reason, and therefore one can now relate that this emotional reason is calm. Furthermore, white was a close second behind blue where the majority chose it because of known medications.

With regards to the stimulant efficacy, both red and yellow-colored pills were the highest voted by participants who attributed their choices primarily to an emotional connection. These colors were highly associated with strength-related traits such as anger, aggression, and power for red, and energy for yellow.

For the anti-anxiety efficacy, participants’ top choice was white, followed by blue, and both were attributed to an emotional reason which we now understand is calm as per Part 2B.

As per the pain relief and antacid efficacies where both had white-colored pills as their most top choice, known medications was the main reason behind participants’ choices. This could be attributed to popular medications Panadol, Aspirin, and Tylenol, for pain relief, and Gaviscon and Alka-Seltzer for antacid [15].

Red was participants’ top choice for the hallucinogenic efficacy, followed closely by yellow. For both choices, the top two reasons for these colors were emotional and symbolic. As mentioned above, red and yellow mainly resemble strength-related attributes and energy.

**Figure 13:**
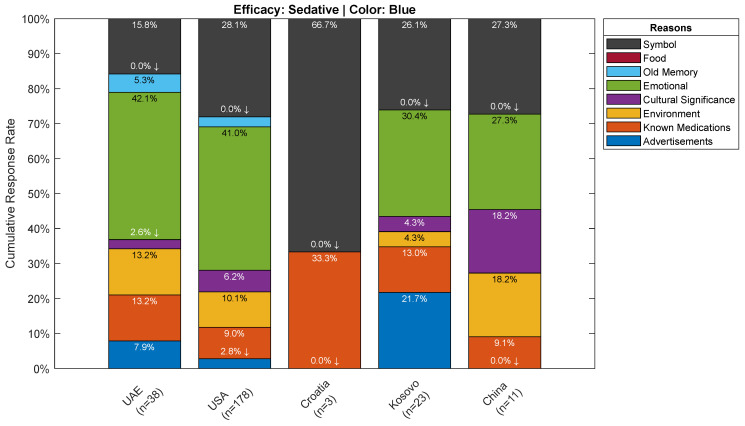
Reasoning Breakdown by Demographical Locations for the participants who selected Blue as most strongly associated with the Sedative Efficacy.

Taking a deeper look at the breakdown of the reasoning results by demographics per efficacy, and its top choice of color, one can observe general trends that are common across demographics. While the two most identified reasons are emotional and symbolic for all efficacies, in addition to known mediations for the pain relief and antacid efficacies, other specific patterns emerge in certain cases such as the one for the location demographic category (See Figure 13). In the case of Croatia, as one example out of many, it stood out as not being influenced by an emotional reason, but rather, symbol is a major factor given that two-thirds of participants attributed their choice of blue as a sedative to it. On the other hand, the remaining participants choose known medications as the reason behind their choice of blue. However, because this breakdown includes reasons only for those who selected blue, this limits the number of observations to small numbers in some groups, including Croatia. Thus, it is advisable not to generalize these results.

#### 3.2.2. Part 2B: Color Association

**Figure 14:**
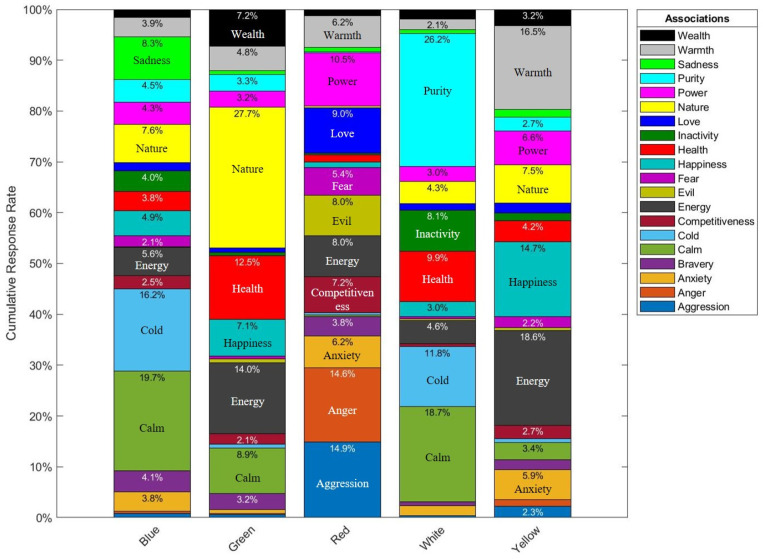
Participants’ response rates showing overall association by color. Labels were placed where space is available.

Looking at Figure 14 below, it is clear that the top word associations with blue were calm at 19.7% and cold at 16.2% response rates respectively. Unsurprisingly, 27.7% of participants associated green with nature. Red was highly associated with aggression at 14.9%, anger at 14.6%, and power at 10.5%. For white, the top choices of participants were purity at 26.2% and calm at 18.7%. Finally, yellow was highly associated with energy at 18.6%, warmth at 16.5%, and happiness at 14.7% response rates. Numerical values less than 2% are not shown in Figure 14.

**Figure 15:**
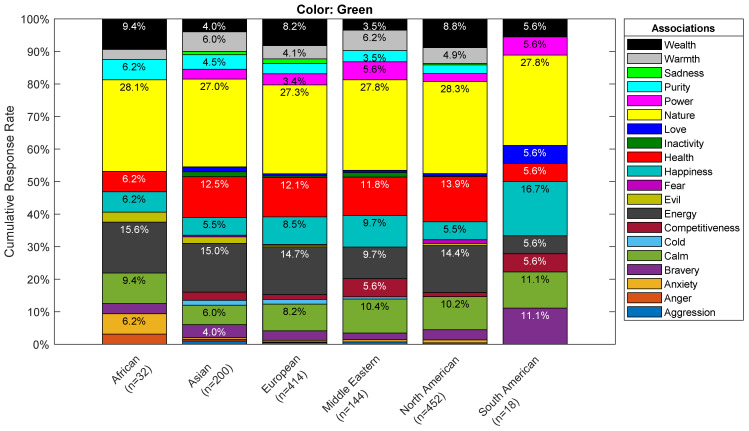
Associations Breakdown by Ethnicity for the Green Color based on number of responses.

Furthermore, the breakdown of associations across demographics demonstrates consistent patterns per color. Figure 15 below shows the associations of green by ethnic groups. One can easily identify that the main association is with nature. The same overall trend was observed for the other demographics as well.

### 3.3. Discussion

The results may be considered in context with previous research, per color. Looking at blue and its association with the word calm as per Part 2B, blue has been constantly associated with calm in the literature as well [19,20,25,28]. This in turn explains its association with the sedative efficacy, and being second highest color for the anti-anxiety efficacy, and the lowest for the stimulant efficacy (See Figure 9).

As for red, Part 2B and previous literature has associated it with activity, stimulations, excitement, anger, aggression, and power [5,19,20,21,22,27,30,31,32]. This in turn explains why red was consistently ranked highest for the efficacies stimulant and hallucinogenic, and ranked lowest for both sedative and anti-anxiety (See Figure 9).

White was mainly associated with both purity and calm as per Part 2B, in addition to prior studies [5,27,29,33]. White ranked most effective for the anti-anxiety, pain relief, and antacid efficacies, and second highest for the sedative efficacy category (See Figure 9).

With regards to yellow, this color was mainly associated with energy and warmth as per Part 2B, which is supporting Ballast’s findings in 2002, Jones’s in 2015, and AL-Ayash’s findings in 2016 [20,25,34]. Yellow was the second top choice for both the stimulant and hallucinogenic efficacies (See Figure 9).

Similar to experiment 1, green did not have any strong association with any of the efficacies, and this again may be due its association with health [27], rather than medications or the efficacies included in this study.

## 4. Summary

Several general trends were observed from both experiments. For the sedative efficacy, blue and white were the first and second-most associated colors. As for the stimulant efficacy, red and yellow were the first and second-most associated colors. With regards to the anti-anxiety efficacy, blue and white were interchangeably the top two choices, and red was the least. For the pain relief efficacy, white was by far the most associated color. As for the antacid efficacy, white and yellow were the first and second-most associated colors, while red and green were the least. Finally, in the hallucinogenic efficacy category, red and yellow were the top two choices of participants in both experiments.

With regards to participants’ color reasoning, the top three most common choices picked by participants were either an emotional reason, a symbol, or known medications. On the other hand, other categories such as food, old memory, cultural significance, environment, and advertisements had lower scores. It is also interesting to note that advertisements scored one of the lowest categories even though one might assume the opposite given the influx of advertisements related to medications in various media channels; perhaps there is an unacknowledged impact that was not recorded. Looking at the sedative, stimulant, and anti-anxiety efficacies, the majority of participants attributed the reasoning behind their choices to an emotional one. As for both the pain relief and the antacid efficacies, the reasoning was attributed to known medications. Finally, emotional and symbolic reasons were what participants attributed their color choices to for the hallucinogenic efficacy.

Generally, the color associations identified by participants were in line with prior research and reported literature. These findings showed us that participants associate blue the most with calm and cold out of the options provided to them in the survey. Also, green with nature, red with aggression, anger, and power, white with purity and calm, and finally yellow with energy, warmth, and happiness.

Finally, future valuable work can include looking at two-colored capsules, more efficacies, and supplements, the possibility of future experiments involving taking placebo pills, a larger range of colors including different shades of the same color. While previous research done by Wan et al. in 2015 [14] reported that brightness had no significant effect on the results, it might be worth exploring in the future, expanding on our current work, with various saturation levels and chroma of the existing colors used in this study. Additionally, prior research [14,35] has demonstrated a correlation between shapes and colors, and therefore, this can be another area to investigate in the future.

## Figures and Tables

**Table 1:** Demographics’ categories provided to participants.

Age	Gender	Ethnicity	Education Level	Pill Usage Frequency
Under 20	Female	African	Below University Level	Rarely
20–39	Male	Asian	Undergraduate	Moderately
40–60		European	Graduate	Daily
Over 60		Middle-Eastern		
		North American		
		South American		

**Table 2:** Pill color input sRGB values and measured CIE 1931 XYZ and CIELAB values.

Color	R	G	B	X	Y	Z	L*	a*	b*	C*_ab_	*h_ab_*
Blue	116	133	195	130	125	283	58	10	−35	36	285°
Green	85	143	65	76	115	43	56	−36	37	52	134°
Red	193	58	60	114	68	24	44	55	32	63	31°
White	255	255	255	464	486	538	100	0	0	0	0°
Yellow	255	247	107	394	454	139	97	−17	68	70	104°

**Table 3:** Survey responses breakdown by demographics.

Demographics		Number of Responses
Location	UAE	339
	USA	538
	Croatia	51
	Kosovo	26
Gender	Male	387
	Female	564
	Prefer not to say ^1^	7
Age Group ^2^	Under 20	200
	20 to 39	412
	Over 39	342
Pill Usage Frequency	Rarely	439
	Moderately	98
	Daily	415
	Prefer not to say ^1^	2
Ethnicity	African	32
	Asian	150
	European	243
	Middle Eastern	100
	North American	411
	South American	18
Educational Level ^2^	Below University Level	94
	Undergraduate	469
	Graduate	391

^1^ Excluded responses from the analysis, ^2^ Contains groups with small sample sizes which were collapsed into other groups.

**Table 4:** Post-hoc test results using Mann–Whitney, Wilcoxon rank-sum, including *p*-values ^1^ and Cohen’s d effect size ^2^.

	Sedative		Stimulant		Anti-Anxiety		Pain Relief		Antacid		Hallucinogenic	
Color 1/Color 2	*p*	d	*p*	d	*p*	d	*p*	d	*p*	d	*p*	d
Blue/Green	**<0.001**	M	**0.001**	VS	**<0.001**	M	**<0.001**	S	**<0.001**	S	0.154	VS
Blue/Red	**<0.001**	S	**<0.001**	L	**<0.001**	L	**<0.001**	S	**<0.001**	S	**<0.001**	S
Blue/White	0.649	VS	0.050	VS	0.873	VS	**<0.001**	S	**<0.001**	VS	**<0.001**	S
Blue/Yellow	**<0.001**	S	**<0.001**	M	**<0.001**	S	**0.016**	VS	**<0.001**	S	**<0.001**	S
Green/Red	0.593	VS	**<0.001**	M	**<0.001**	M	**0.006**	VS	**0.014**	VS	**<0.001**	VS
Green/White	**<0.001**	S	0.642	VS	**<0.001**	S	**<0.001**	M	**<0.001**	S	**<0.001**	S
Green/Yellow	**<0.001**	S	**<0.001**	S	**<0.001**	S	**<0.001**	S	**<0.001**	S	**<0.001**	S
Red/White	**<0.001**	S	**<0.001**	M	**<0.001**	L	**<0.001**	M	**<0.001**	S	**<0.001**	M
Red/Yellow	**<0.001**	VS	**<0.001**	S	**<0.001**	L	**0.024**	VS	**<0.001**	M	0.958	VS
White/Yellow	**<0.001**	S	**<0.001**	S	**<0.001**	S	**<0.001**	S	0.852	VS	**<0.001**	M

^1^ Red text indicates significant differences (alpha = 0.05) after Holms-Bonferroni correction. ^2^ Letters indicate Sawilowsky’s categorization of Cohen’s d effect sizes: Very Small (VS), Small (S), Medium (M), Large (L), Very Large (VL), Huge (H). Otherwise, No Improvement (NI).

**Table 5:** KW statistical test results for each ethnicity and efficacy category.

	Sedative		Stimulant		Anti-Anxiety		Pain Relief		Antacid		Hallucinogenic	
Ethnicity	X^2^	*p*	X^2^	*p*	X^2^	*p*	X^2^	*p*	X^2^	*p*	X^2^	*p*
African	7.99	0.092	16.65	**0.0023**	7.92	0.0947	9.23	0.0556	11.6	**0.0206**	11.51	**0.0214**
Asian	11.3	**0.0234**	55.95	**<0.001**	30.35	**<0.001**	20.23	**0.0005**	9.93	**0.0417**	25.98	**<0.001**
European	146.62	**<0.001**	148.5	**<0.001**	236.41	**<0.001**	97.31	**<0.001**	47.39	**<0.001**	61.15	**<0.001**
Middle Eastern	6.56	0.1608	61.17	**<0.001**	27.72	**<0.001**	28.31	**<0.001**	11.39	**0.0225**	13.96	**0.0074**
North American	142.23	**<0.001**	213.37	**<0.001**	375.51	**<0.001**	152.13	**<0.001**	168.84	**<0.001**	87.9	**<0.001**
South American	2.93	0.5699	6.4	0.1713	12.2	**0.0159**	7.82	0.0983	20.53	**0.0004**	1.21	0.8771

Red text indicates significant effects (alpha ≤ 0.05).

**Table 6:** KW statistical test results for each age and efficacy category.

	Sedative		Stimulant		Anti-Anxiety		Pain Relief		Antacid		Hallucinogenic	
Age Bracket	X^2^	*p*	X^2^	*p*	X^2^	*p*	X^2^	*p*	X^2^	*p*	X^2^	*p*
Under 20	41.76	**<0.001**	96.15	**<0.001**	99.71	**<0.001**	49.09	**<0.001**	21.7	**0.0002**	22	**0.0002**
20 to 39	89.51	**<0.001**	167.19	**<0.001**	294.09	**<0.001**	140.95	**<0.001**	46.02	**<0.001**	84.43	**<0.001**
Over 39	146	**<0.001**	184.27	**<0.001**	249.62	**<0.001**	119.18	**<0.001**	214.22	**<0.001**	114.02	**<0.001**

Red text indicates significant effects (alpha ≤ 0.05).

**Table 8:** List of reasons provided to participants.

Advertisements—Branding and marketing campaigns on TV, radio, or social media
Known Medications—Previous and/or current knowledge of similar drugs
Environment—Similarity to landscape and/or nature
Cultural Significance—Influenced by cultural or traditional norms
Emotional—The way it makes me feel
Old Memory—Reminds me of a previous memory/connection
Food—First food thought that comes to mind
Symbol—What this color represents to me personally

**Table 9:** Survey responses breakdown by demographics.

Demographics		Number of Responses
Location	UAE	109
	USA	232
	Croatia	18
	Kosovo	35
	China	16
Gender	Males	170
	Females	240
Age Group	Under 20	73
	20 to 39	164
	40 to 60	117
	Over 60	56
Pill Usage Frequency	Rarely	182
	Moderately	59
	Daily	169
Ethnicity	African	14
	Asian	69
	European	131
	Middle Eastern	48
	North American	143
	South American	5
Educational Level	Below University Level	23
	Undergraduate	175
	Graduate	212

## Data Availability

All data described in this study are available upon reasonable request.

## Appendix A

**Table A1:** Post hoc Mann–Whitney, Wilcoxon rank-sum test results by ethnicity and efficacy, including *p*-values ^1^ and interpreted Cohen’s d effect size ^2^.

	Sedative												Stimulant											
	African		Asian		European		Middle Eastern		North American		South American		African		Asian		European		Middle Eastern		North American		South American	
Color 1/Color 2	*p*	d	*p*	d	*p*	d	*p*	d	*p*	d	*p*	d	*p*	d	*p*	d	*p*	d	*p*	d	*p*	d	*p*	d
Blue/Green	-	-	**0.003**	S	**<0.001**	L	-	-	**<0.001**	M	-	-	0.954	VS	0.106	VS	0.355	VS	0.371	VS	**0.002**	S	-	-
Blue/Red	-	-	0.568	VS	**<0.001**	L	-	-	**<0.001**	M	-	-	**0.003**	M	**<0.001**	M	**<0.001**	L	**<0.001**	M	**<0.001**	L	-	-
Blue/White	-	-	0.824	VS	0.501	VS	-	-	0.938	VS	-	-	0.106	S	0.865	NI	0.746	VS	**<0.001**	M	**<0.001**	S	-	-
Blue/Yellow	-	-	0.265	VS	**<0.001**	M	-	-	**<0.001**	S	-	-	0.011	M	**0.005**	S	**<0.001**	M	0.639	VS	**<0.001**	M	-	-
Green/Red	-	-	**0.005**	S	0.078	VS	-	-	0.449	VS	-	-	**0.001**	L	**<0.001**	M	**<0.001**	L	**<0.001**	S	**<0.001**	M	-	-
Green/White	-	-	0.056	S	**<0.001**	M	-	-	**<0.001**	M	-	-	0.096	S	0.249	VS	0.633	VS	**<0.001**	M	**0.046**	VS	-	-
Green/Yellow	-	-	0.059	S	**0.005**	S	-	-	**<0.001**	S	-	-	0.007	M	0.297	VS	**<0.001**	M	0.627	VS	**<0.001**	M	-	-
Red/White	-	-	0.436	VS	**<0.001**	M	-	-	**<0.001**	M	-	-	0.195	S	**<0.001**	M	**<0.001**	L	**<0.001**	L	**<0.001**	M	-	-
Red/Yellow	-	-	0.146	VS	**<0.001**	S	-	-	**<0.001**	S	-	-	0.412	VS	**<0.001**	S	**<0.001**	S	**<0.001**	S	**<0.001**	VS	-	-
White/Yellow	-	-	0.528	VS	**<0.001**	S	-	-	**<0.001**	S	-	-	0.492	VS	0.056	S	**<0.001**	M	**<0.001**	M	**<0.001**	S	-	-
	**Anti-Anxiety**												**Pain Relief**											
	**African**		**Asian**		**European**		**Middle** **Eastern**		**North** **American**		**South** **American**		**African**		**Asian**		**European**		**Middle** **Eastern**		**North** **American**		**South** **American**	
**Color 1/Color 2**	***p***	**d**	***p***	**d**	***p***	**d**	***p***	**d**	***p***	**d**	***p***	**d**	***p***	**d**	***p***	**d**	***p***	**d**	***p***	**d**	***p***	**d**	***p***	**d**
Blue/Green	-	-	0.026	S	**<0.001**	L	**0.003**	S	**<0.001**	M	0.052	M	-	-	0.372	VS	**0.000**	M	**0.000**	M	**0.000**	S	-	-
Blue/Red	-	-	**<0.001**	M	**<0.001**	VL	**<0.001**	M	**<0.001**	VL	0.013	L	-	-	0.766	VS	**0.000**	S	0.030	S	0.033	VS	-	-
Blue/White	-	-	0.949	VS	0.405	VS	0.952	VS	0.550	NI	0.968	NI	-	-	**0.001**	S	**0.000**	S	0.074	VS	**0.000**	S	-	-
Blue/Yellow	-	-	0.313	VS	**<0.001**	M	0.069	S	**<0.001**	S	0.074	M	-	-	0.526	VS	0.027	VS	0.040	S	0.149	VS	-	-
Green/Red	-	-	**0.002**	S	**<0.001**	M	**0.006**	S	**<0.001**	M	0.252	S	-	-	0.706	VS	0.471	VS	0.620	VS	**0.005**	S	-	-
Green/White	-	-	0.050	VS	**<0.001**	M	0.025	S	**<0.001**	M	0.056	M	-	-	**0.000**	S	**0.000**	L	**0.000**	M	**0.000**	L	-	-
Green/Yellow	-	-	0.128	VS	0.057	VS	0.324	VS	**<0.001**	S	0.709	VS	-	-	0.128	VS	**0.000**	S	0.026	S	**0.000**	S	-	-
Red/White	-	-	**<0.001**	S	**<0.001**	VL	**<0.001**	M	**<0.001**	VL	0.008	L	-	-	**0.001**	S	**0.000**	M	**0.002**	S	**0.000**	M	-	-
Red/Yellow	-	-	**<0.001**	S	**<0.001**	L	**0.001**	S	**<0.001**	L	0.128	M	-	-	0.410	VS	0.048	S	0.350	VS	0.301	VS	-	-
White/Yellow	-	-	0.340	VS	**<0.001**	S	0.184	VS	**<0.001**	S	0.126	M	-	-	**0.003**	S	**0.000**	M	**0.001**	S	**0.000**	S	-	-
	**Antacid**												**Hallucinogenic**											
	**African**		**Asian**		**European**		**Middle** **Eastern**		**North** **American**		**South** **American**		**African**		**Asian**		**European**		**Middle** **Eastern**		**North** **American**		**South** **American**	
**Color 1/Color 2**	***p***	**d**	***p***	**d**	***p***	**d**	***p***	**d**	***p***	**d**	***p***	**d**	***p***	**d**	***p***	**d**	***p***	**d**	***p***	**d**	***p***	**d**	***p***	**d**
Blue/Green	0.560	VS	0.895	VS	**0.006**	S	0.704	VS	**<0.001**	S	0.698	VS	0.215	S	0.520	VS	0.453	VS	0.682	VS	0.484	VS	0.574	VS
Blue/Red	0.703	VS	0.919	NI	0.047	S	0.092	S	**<0.001**	M	0.051	M	0.502	VS	0.013	S	**0.003**	S	0.335	VS	**<0.001**	S	0.305	S
Blue/White	0.011	M	0.120	VS	**0.009**	VS	0.009	S	**<0.001**	S	0.074	M	0.229	S	0.085	VS	**<0.001**	S	**0.007**	S	**<0.001**	S	0.497	S
Blue/Yellow	0.028	M	0.007	S	**0.002**	S	0.619	VS	**0.001**	S	0.012	L	**0.005**	M	**<0.001**	S	**0.004**	S	0.479	VS	**<0.001**	S	0.698	VS
Green/Red	0.861	NI	0.845	VS	0.876	VS	0.231	VS	**0.002**	VS	0.117	M	0.553	VS	0.099	VS	0.028	VS	0.599	VS	**0.003**	VS	0.575	S
Green/White	0.019	M	0.093	VS	**<0.001**	S	0.035	S	**<0.001**	M	0.042	M	0.096	S	0.024	S	**<0.001**	S	**0.004**	S	**<0.001**	S	0.847	VS
Green/Yellow	0.049	S	0.009	S	**<0.001**	M	0.402	VS	**<0.001**	M	0.006	L	0.222	S	**0.006**	S	0.041	VS	0.852	VS	**0.007**	VS	0.954	NI
Red/White	0.033	M	0.171	VS	**<0.001**	S	0.435	VS	**<0.001**	M	**0.002**	VL	0.154	S	**0.001**	S	**<0.001**	M	**0.003**	S	**<0.001**	M	0.831	VS
Red/Yellow	0.100	S	0.047	S	**<0.001**	S	0.059	S	**<0.001**	M	**<0.001**	VL	0.049	M	0.527	VS	0.685	VS	0.591	VS	0.632	NI	0.575	S
White/Yellow	0.519	VS	0.805	VS	0.893	VS	0.005	S	0.098	VS	0.659	S	0.006	L	**<0.001**	M	**<0.001**	M	**0.003**	S	**<0.001**	M	0.742	VS

^1^ Red text indicates significant differences (alpha ≤ 0.05) after Holms-Bonferroni correction. ^2^ Letters indicate Sawilowsky’s categorization of Cohen’s d effect sizes: Very Small (VS), Small (S), Medium (M), Large (L), Very Large (VL), Huge (H). Otherwise, No Improvement (NI).

**Table A2:** Post hoc Mann–Whitney, Wilcoxon rank-sum test results by age and efficacy, including *p*-values ^1^ and interpreted Cohen’s d effect size ^2^.

	Sedative						Stimulant						Anti-Anxiety					
	Under 20		20 to 39		Over 39		Under 20		20 to 39		Over 39		Under 20		20 to 39		Over 39	
Color 1/Color 2	*p*	d	*p*	d	*p*	d	*p*	d	*p*	d	*p*	d	*p*	d	*p*	d	*p*	d
Blue/Green	**<0.001**	M	**<0.001**	M	**<0.001**	M	0.120	VS	0.052	VS	0.035	VS	**<0.001**	M	**<0.001**	M	**<0.001**	M
Blue/Red	0.011	S	**<0.001**	S	**<0.001**	M	**<0.001**	L	**<0.001**	M	**<0.001**	L	**<0.001**	L	**<0.001**	L	**<0.001**	L
Blue/White	0.124	S	0.751	VS	0.461	NI	0.834	VS	0.364	VS	0.039	VS	0.407	VS	0.865	VS	0.484	VS
Blue/Yellow	**<0.001**	S	**<0.001**	S	**<0.001**	S	**<0.001**	M	**<0.001**	S	**<0.001**	M	**<0.001**	S	**<0.001**	S	**0.001**	S
Green/Red	0.010	S	0.021	S	**<0.001**	S	**<0.001**	M	**<0.001**	M	**<0.001**	M	**<0.001**	S	**<0.001**	M	**<0.001**	M
Green/White	**<0.001**	S	**<0.001**	M	**<0.001**	M	0.324	VS	0.601	VS	0.615	VS	**<0.001**	S	**<0.001**	S	**<0.001**	S
Green/Yellow	0.050	VS	**<0.001**	S	**<0.001**	S	**<0.001**	S	**<0.001**	S	**<0.001**	M	**0.014**	S	**0.017**	VS	**<0.001**	S
Red/White	0.481	VS	**0.002**	S	**<0.001**	M	**<0.001**	M	**<0.001**	M	**<0.001**	M	**<0.001**	M	**<0.001**	L	**<0.001**	L
Red/Yellow	0.242	VS	0.786	VS	**<0.001**	M	**<0.001**	S	**<0.001**	S	**<0.001**	VS	**<0.001**	M	**<0.001**	M	**<0.001**	L
White/Yellow	0.010	S	**<0.001**	S	**<0.001**	S	**<0.001**	S	**<0.001**	S	**<0.001**	M	**0.004**	S	**<0.001**	S	**<0.001**	S
	**Pain Relief**						**Antacid**						**Hallucinogenic**					
	**Under 20**		**20 to 39**		**Over 39**		**Under 20**		**20 to 39**		**Over 39**		**Under 20**		**20 to 39**		**Over 39**	
**Color 1/Color 2**	***p***	**d**	***p***	**d**	***p***	**d**	***p***	**d**	***p***	**d**	***p***	**d**	***p***	**d**	***p***	**d**	***p***	**d**
Blue/Green	**<0.001**	S	**<0.001**	S	**<0.001**	S	0.224	VS	**0.003**	S	**<0.001**	S	0.537	VS	**<0.001**	S	**0.032**	VS
Blue/Red	0.112	S	0.136	VS	**<0.001**	S	0.743	VS	0.320	VS	**<0.001**	M	0.186	VS	**0.012**	VS	**<0.001**	S
Blue/White	**0.004**	S	**<0.001**	S	**<0.001**	S	0.787	VS	**0.008**	VS	**<0.001**	S	0.016	S	**<0.001**	S	**<0.001**	S
Blue/Yellow	0.042	VS	0.023	VS	0.993	VS	**<0.001**	S	**0.002**	S	**0.002**	S	0.013	S	**<0.001**	S	**0.021**	VS
Green/Red	0.041	S	**0.001**	S	0.676	NI	0.511	VS	0.128	VS	**<0.001**	S	0.480	VS	0.313	VS	**<0.001**	S
Green/White	**<0.001**	M	**<0.001**	L	**<0.001**	M	0.239	VS	**<0.001**	S	**<0.001**	M	**0.005**	S	**<0.001**	M	**<0.001**	S
Green/Yellow	**0.003**	S	**<0.001**	S	**<0.001**	S	**<0.001**	S	**<0.001**	S	**<0.001**	M	0.083	VS	0.335	VS	**<0.001**	S
Red/White	**<0.001**	S	**<0.001**	S	**<0.001**	M	0.696	VS	**0.001**	S	**<0.001**	L	**0.001**	S	**<0.001**	S	**<0.001**	M
Red/Yellow	0.966	VS	0.832	VS	**<0.001**	S	**0.001**	S	**<0.001**	S	**<0.001**	L	0.368	VS	0.045	VS	**0.006**	VS
White/Yellow	**<0.001**	S	**<0.001**	M	**<0.001**	S	0.008	S	0.998	VS	**0.012**	VS	**<0.001**	S	**<0.001**	M	**<0.001**	M

^1^ Red text indicates significant differences (alpha ≤ 0.05) after Holms-Bonferroni correction. ^2^ Letters indicate Sawilowsky’s categorization of Cohen’s d effect sizes: Very Small (VS), Small (S), Medium (M), Large (L), Very Large (VL), Huge (H). Otherwise, No Improvement (NI).

**Table A3:** Post hoc Mann–Whitney, Wilcoxon rank-sum test results by location and efficacy, including *p*-values^1^ and interpreted Cohen’s d effect size ^2^.

	Sedative								Stimulant								Anti-Anxiety							
	UAE		USA		Croatia		Kosovo		UAE		USA		Croatia		Kosovo		UAE		USA		Croatia		Kosovo	
Color 1/Color 2	*p*	d	*p*	d	*p*	d	*p*	d	*p*	d	*p*	d	*p*	d	*p*	d	*p*	d	*p*	d	*p*	d	*p*	d
Blue/Green	**<0.001**	S	**<0.001**	M	**0.002**	M	-	-	0.927	VS	**<0.001**	S	0.381	VS	0.271	S	**<0.001**	S	**<0.001**	M	**<0.001**	L	**0.001**	L
Blue/Red	0.388	VS	**<0.001**	M	0.064	S	-	-	**<0.001**	M	**<0.001**	L	0.043	S	**<0.001**	VL	**<0.001**	M	**<0.001**	VL	**0.002**	M	**<0.001**	H
Blue/White	0.868	VS	0.614	VS	0.929	VS	-	-	0.163	VS	**<0.001**	S	0.122	S	0.256	S	0.891	VS	0.930	VS	0.948	VS	0.509	VS
Blue/Yellow	0.143	VS	**<0.001**	S	**<0.001**	M	-	-	0.041	S	**<0.001**	M	0.217	S	0.035	M	**0.001**	S	**<0.001**	S	**0.004**	M	0.163	S
Green/Red	**0.002**	S	0.222	VS	0.851	VS	-	-	**<0.001**	M	**<0.001**	M	0.009	M	**0.001**	L	**<0.001**	S	**<0.001**	M	0.761	VS	**0.002**	L
Green/White	**<0.001**	S	**<0.001**	M	0.017	S	-	-	0.119	VS	0.394	VS	0.458	VS	0.073	S	**<0.001**	S	**<0.001**	M	0.011	M	**0.001**	L
Green/Yellow	**0.001**	S	**<0.001**	S	0.520	VS	-	-	0.048	S	**<0.001**	M	0.047	S	0.348	S	0.615	VS	**<0.001**	S	0.307	S	0.051	M
Red/White	0.735	VS	**<0.001**	S	0.218	S	-	-	**<0.001**	M	**<0.001**	M	**0.005**	M	**<0.001**	VL	**<0.001**	M	**<0.001**	L	0.020	S	**<0.001**	H
Red/Yellow	0.586	VS	**<0.001**	S	0.510	VS	-	-	**<0.001**	S	**<0.001**	S	0.188	VS	0.007	M	**<0.001**	S	**<0.001**	L	0.305	VS	**<0.001**	VL
White/Yellow	0.205	VS	**<0.001**	S	0.007	M	-	-	**0.002**	S	**<0.001**	S	0.021	S	0.012	M	**0.001**	S	**<0.001**	S	0.050	S	0.071	S
	**Pain Relief**								**Antacid**								**Hallucinogenic**							
	**UAE**		**USA**		**Croatia**		**Kosovo**		**UAE**		**USA**		**Croatia**		**Kosovo**		**UAE**		**USA**		**Croatia**		**Kosovo**	
**Color 1/Color 2**	***p***	**d**	***p***	**d**	***p***	**d**	***p***	**d**	***p***	**d**	***p***	**d**	***p***	**d**	***p***	**d**	***p***	**d**	***p***	**d**	***p***	**d**	***p***	**d**
Blue/Green	**0.001**	S	**<0.001**	S	0.017	S	0.174	S	0.196	VS	**<0.001**	S	-	-	-	-	0.456	VS	0.184	VS	0.997	NI	-	-
Blue/Red	**0.002**	S	0.034	VS	0.053	S	0.010	M	0.022	S	**<0.001**	S	-	-	-	-	0.043	VS	**<0.001**	VS	0.017	S	-	-
Blue/White	**<0.001**	S	**<0.001**	S	0.277	VS	0.014	M	0.607	VS	**<0.001**	S	-	-	-	-	0.055	VS	**<0.001**	S	0.011	S	-	-
Blue/Yellow	0.089	VS	0.150	VS	0.065	S	0.820	VS	0.022	S	**<0.001**	S	-	-	-	-	0.008	S	**<0.001**	S	0.094	S	-	-
Green/Red	0.457	VS	**<0.001**	S	0.969	VS	0.129	S	0.274	VS	**0.004**	VS	-	-	-	-	0.285	VS	**0.003**	VS	0.042	S	-	-
Green/White	**<0.001**	M	**<0.001**	M	**0.001**	M	**0.002**	L	0.110	VS	**<0.001**	S	-	-	-	-	0.010	S	**<0.001**	S	0.025	S	-	-
Green/Yellow	0.094	VS	**<0.001**	S	0.403	VS	0.112	S	**0.001**	S	**<0.001**	M	-	-	-	-	0.110	VS	**0.001**	VS	0.201	S	-	-
Red/White	**<0.001**	M	**<0.001**	S	0.007	M	**<0.001**	L	0.018	S	**<0.001**	M	-	-	-	-	**0.001**	S	**<0.001**	M	**<0.001**	L	-	-
Red/Yellow	0.060	VS	0.355	VS	0.607	VS	0.006	L	**<0.001**	S	**<0.001**	M	-	-	-	-	0.816	VS	0.969	VS	0.455	VS	-	-
White/Yellow	**<0.001**	M	**<0.001**	S	**0.003**	M	0.018	S	0.194	VS	0.355	VS	-	-	-	-	**<0.001**	S	**<0.001**	M	**<0.001**	M	-	-

^1^ Red text indicates significant differences (alpha ≤ 0.05) after Holms-Bonferroni correction. ^2^ Letters indicate Sawilowsky’s categorization of Cohen’s d effect sizes: Very Small (VS), Small (S), Medium (M), Large (L), Very Large (VL), Huge (H). Otherwise, No Improvement (NI).
